# How does video case-based learning influence clinical decision-making by midwifery students? An exploratory study

**DOI:** 10.1186/s12909-020-1969-0

**Published:** 2020-03-06

**Authors:** Kana Nunohara, Rintaro Imafuku, Takuya Saiki, Susan M. Bridges, Chihiro Kawakami, Koji Tsunekawa, Masayuki Niwa, Kazuhiko Fujisaki, Yasuyuki Suzuki

**Affiliations:** 1 Medical Education Development Center, Gifu University, Yanagido 1-1, Gifu, 501-1194 Japan; 2 Nursing Department, Gifu College of Nursing, Egira-cho 3047-1, Hashima, Gifu, 501-6295 Japan; 3 Faculty of Education, The University of Hong Kong, Pokfulam Road, Pok Fu Lam, Hong Kong

**Keywords:** Case-based learning, Case modality, Video case, Paper case, Clinical decision-making, Bio-psychosocial consideration

## Abstract

**Background:**

Clinical decision-making skills are essential for providing high-quality patient care. To enhance these skills, many institutions worldwide use case-based learning (CBL) as an educational strategy of pre-clinical training. However, to date, the influence of different learning modalities on students’ clinical decision-making processes has not been fully explored. This study aims to explore the influence of video and paper case modalities on the clinical decision-making process of midwifery students during CBL.

**Methods:**

CBL involving a normal pregnant woman was provided for 45 midwifery students. They were divided into 12 groups; six groups received the video modality, and six groups received the paper modality. Group discussions were video-recorded, and focus groups were conducted after the CBL. Transcripts of the group discussions were analysed in terms of their interaction patterns, and focus groups were thematically analysed based on the three-stage model of clinical decision-making, which includes cue acquisition, interpretation, and evaluation/decision-making.

**Results:**

The students in the video groups paid more attention to psychosocial than biomedical aspects and discussed tailored care for the woman and her family members. They refrained from vaginal examinations and electric fetal heart monitoring. Conversely, the students in the paper groups paid more attention to biomedical than psychosocial aspects and discussed when to perform vaginal examinations and electric fetal heart monitoring.

**Conclusion:**

This study clarified that video and paper case modalities have different influences on learners’ clinical decision-making processes. Video case learning encourages midwifery students to have a woman- and family-centred holistic perspective of labour and birth care, which leads to careful consideration of the psychosocial aspects. Paper case learning encourages midwifery students to have a healthcare provider-centred biomedical perspective of labour and childbirth care, which leads to thorough biomedical assessment.

## Background

Current midwifery educators are finding it increasingly difficult to provide adequate opportunities for students to learn how to offer effective care for women with normal pregnancies in clinical placements [1, 2]. Several factors impact this issue: the increased risk(s) of interventions for pregnant women over the age of 35 [3], societal awareness of women’s rights and concerns about litigation [4, 5], and decreased birth rates [6]. As health professionals, midwives are responsible for making important clinical decisions regarding positive childbirth experiences for pregnant women. Thus, midwifery students must acquire clinical decision-making skills that effectively support pregnant women and their family members from both physical and psychosocial perspectives.

Due to the sharply declining trend of birth rates in Japan (e.g. 1.42 in 2018), midwifery students have fewer learning opportunities involving normal pregnancies. Ninety-nine percent of Japanese women give birth with assistance from midwives in maternity hospitals in collaboration with obstetricians; only 1% give birth in midwifery homes. Fifty-eight percent of births occur in the presence of the women’s partners for emotional support [7].

Midwifery students participate in either a one-year program at an independent midwifery school after completing a 3 to 4-year nursing program or a 1 to 1.5-year midwifery course within a 4-year nursing program. In general, while some midwifery students have clinical experience as registered nurses before receiving their midwifery education, most lack any such experience.

Case-based learning (CBL) is an inquiry-based pedagogical approach that prepares students for clinical practice through authentic cases to develop their clinical decision-making skills. It has been implemented in various pre-clinical and clinical settings [8, 9]. CBL before clinical placement is a common educational method in Japan, used in more than 70% of midwifery schools [10]. Successful implementation of CBL can foster students’ learning in terms of knowledge acquisition and application, intrinsic motivation [8], patient assessment [11], problem-solving [12], and critical thinking [13, 14]. Moreover, pictorial information in the case materials induces intuition, and objective and quantitative information induces analysis [15].

Previous studies have revealed that students tend to prefer video cases since they perceive video modality as authentic [16, 17], interesting [18], motivating [19], and stimulating [20]. Video cases elicit students’ attention and emotions [21], promote empathy [22], improve memory retention [17], increase understanding of the cases [18], and improve students’ patient-centredness [23]. However, some studies have questioned whether video cases make it difficult to identify relevant information and also hamper information retention [24] and deep critical thinking [20]. Two-thirds of students preferred paper cases since video cases impeded their ability to critically review the presented information [24]. Moreover, some students perceived that the written texts of case materials were more reliable since they could reread the contents and learn at their own pace [25].

Despite these studies, the influences of case modalities on students’ learning processes for clinical decision-making have yet to be thoroughly explored. In particular, although clinical decision-making skills are essential to providing high-quality patient care, they have not been fully examined in the field of nurse and midwifery education.

 This study aims to explore the influences of video and paper case modalities on the clinical decision-making processes of midwifery students during CBL. These two modalities were chosen based on their high feasibility and popularity in the world.

Using a three-stage model of clinical decision-making developed based on the concept of hypothetical deductive reasoning [26, 27], we analysed the following characteristics of learning modalities in CBL:

1. Cue acquisition: acquisition and retention of clues necessary to interpret the case information.
2. Interpretation: interpretation and understanding of the obtained clues in the case information.
3. Evaluation/decision-making: selection of an optimum plan and related decision-making based on the understanding of the case information.

## Methods

### Case development

First, we developed a typical paper case regarding a full-term primipara who experienced a normal birth in a maternity hospital based on the ‘Evidence-based Midwifery Guidelines’ published by the Japan Academy of Midwifery [28]. The case consisted of six common scenes of interventions by midwives from the primipara’s admission to the maternity hospital with her partner to the time that she entered the birthing room (Table 1): admission (Scene 1), after the morning rounds by an obstetrician (Scene 2), after lunch and once moderate labour pain occurs every 5 min (Scene 3), once labour pain extends to the perianal region indicating the need for a vaginal examination (VE) (Scene 4), once labour pain and anal compression have increased (Scene 5), and typical features of the second stage (Scene 6). At Scene 5, the midwife should confirm the progress of labour and focus on the psychological status of the woman. This scenario includes common clinical problems that midwifery students might encounter in clinical sites, information regarding the labour progress, and the conversations between the woman and her family members. Facial expressions and emotions of the woman and her family are also described in the paper scenario.

**Table 1:** Case overview

Stage of labour		Scene (time)	Labour progress	Situation of pregnant woman and her family	Discussion time (video clip)
First stage	Latent phase	1. Admission (5 AM)	Mild labour pain every 8 min 3 cm dilatation	A pregnant woman was hospitalized, accompanied by her partner. She had positive feelings toward childbirth and was slightly excited, and she talked about how she was at home.	25′ (4′10″)
		2. After an obstetrician’s consultation (9 AM)	Mild labour pain every 8 min 3 cm dilatation	VE by an obstetrician showed little change after hospitalization. Her partner was somewhat discouraged. He told her that he would like to go to work if she is not going to be born soon.	18′ (2′12″)
	Active phase	3. After lunch (1 PM)	Moderate labour pain every 5 min 4 cm dilatation	She was tired and looked sleepy. Her mother insisted that she not sleep since the labour would be delayed if she fell asleep.	15′ (1′49″)
		4. Snack time (3 PM)	Moderate labour pain every 3–5 min 6 cm dilatation	She complained of lower back pain and mild perianal region. Her partner explained the labour situation to the midwife. The patient could not eat a snack because of labour pain.	10′ (1′17″)
		5. Increased labour pain (5 PM)	Strong labour pain every 3 min 8 cm dilatation	She complained of a feeling of anal compression and said “I am afraid of labour, I may not be able to bear it”. Her partner became embarrassed. She became frustrated because her mother told her “try a little harder to do your best.”	15′ (1′51″)
Second stage		6. Having an urge to push (6 PM)	Strong labour pain every 3 min 10 cm full dilatation	Pushing and water breaking occurred. She asked, “Shouldn’t I push yet?” and complained urgently “I feel something has flowed out.”	15′ (1′57″)

A video case was precisely reproduced from the paper scenario. Professional actors played the roles of the woman and her partner under the instruction of midwife teachers using video learning material. The midwife teachers played the roles of the woman’s mother and a midwife in the video. The authenticity and consistency regarding the textual and visual descriptions of the labour processes in the paper and video cases were reviewed by four experts in midwifery, each with a minimum of 8 years of clinical experience.

### Participants

Three midwifery schools (A, B, and C) in the areas surrounding the researchers’ institutions were selected as the research sites. Fifty midwifery students were invited to participate, and consent was obtained from 45 students (90.0%). All participants had completed at least 3 years of a nursing program and were taking a one-year, full-time midwifery course. At the time of their participation, they had completed the four-month pre-clinical midwifery program (which focused on childbirth care, midwifery philosophy, and women-centred care) and were about to begin their clinical placements. Nine of 45 students (20.0%) had worked as registered nurses after graduating from nursing schools before entering the midwifery program; the other 36 (80%) had no clinical work experience as registered nurses and were taking the midwifery course directly after completing nursing school.

The participants were assigned to 12 similar small groups based on information from the midwifery teachers, such as their age, previous learning experience, academic achievement, nursing experience, commutation skills, and (if possible) their own childbirth experience. The number of students per group was limited to four (maximum five) to maximize student engagement in the CBL. Six groups were assigned to video case learning (V1–V6: three groups from School A, one from School B, and two from School C for a total of 24 students), and six were assigned to paper case learning (P1–P6: four groups from School A, one from School B, and one from School C for a total of 21 students). All students were female with an average age of 24.5 years (21 to 41 years) for the video groups and 24.1 years (21 to 41 years) for the paper groups.

### Intervention

The CBLs were conducted as extracurricular activities in Japanese. The researchers (KN, CK, and RI) prepared the classroom setting, recorded and managed timekeeping, and allowed the students to discuss their experiences in Japanese. However, they did not facilitate the students’ discussions in order to gather data in a natural setting. Prior to the CBL activities, the students in both groups were asked to read the background information on the case for 5 min, including the woman’s profile, labour progress, and birth plan. During the CBL activities, the researchers provided a paper scenario or replayed a video clip for each scene in a stepwise manner. After reading or watching each scene with the group members, the students were asked to perform the following tasks: 1) assess the woman and her family members, 2) create a care plan, and 3) decide on an action plan. The times allotted for group discussions of each scene are shown in Table 1. The students received additional information regarding the VE and/or electric fetal heart monitoring (EFM) when they decided to perform VE or EFM in each scene.

### Data collection

All group discussions during the CBL activities were video- and audio-recorded. Following such activities, focus groups were conducted by KN, CK and RI in order to elicit reflective data, such as the participants’ perceptions of the learning process, any difficulties that they encountered, and the influence(s) of the case modality (i.e., video or paper) on their learning. The focus groups were conducted in Japanese and then translated into English by the researchers.

### Ethical considerations

This research was approved by the Institutional Review Board of the Gifu University Graduate School of Medicine (No. 24–366). The students were asked to participate in this study several weeks before conducting the CBL activities so that they were given enough time to make an informed decision. Moreover, the researcher emphasized that the study was completely voluntary and that the participants could withdraw at any time, without negative consequences. Finally, the participants were asked to mail their consent forms directly and individually to the researchers to preserve their anonymity. None of the faculty members at any of the schools attended the sessions when the researchers explained the project, and none of the researchers were engaged as faculty members at the three schools.

### Data analysis

To interpret and describe the influences of the case modalities on participants’ clinical decision-making processes, both focus groups and recorded data from group discussions during CBL were analysed using qualitative content analysis [29, 30]. Qualitative content analysis is a systematic and flexible method for describing the meaning of qualitative data through reducing the amount of material [31]. The text materials for qualitative content analysis include all sorts of recorded communication such as transcripts of interviews, discourses, protocols of observations, and videos [29]. Adopting this analytical approach, this study developed the categories of coding frame in terms of the students’ perceptions of and experiences with CBL.

Codes were iteratively developed during the coding process in addition to those developed from the three-stage model of clinical decision-making. This theoretical consideration led to developing further categories or rephrasing/revising the categories [30]. The steps of coding include selecting material, structuring and generating categories based on theory or previous studies, defining categories, and revising and expanding the frame [31].

Following an independent qualitative content analysis of focus group transcripts by KN, RI, TS, and YS, the overlying themes and subcategories were cross-checked, and the findings were carefully reviewed by all of the researchers at the stages of defining, revising, and expanding the frame. Coding disagreements were resolved through discussion leading to refinement of the coding frame.

The transcripts of the group discussions were also analysed using qualitative content analysis with a method of process coding. In the process coding, simple observable activity (e.g. questioning, answering, agreeing, and acknowledging) and more general conceptual action (e.g. struggling, negotiating, adapting, and applying) can be coded to each exchange in the conversation [32]. Employing the process coding, the distinctive exchange patterns of the video and paper groups were extracted by four independent researchers (KN, RI, TS, and YS). Furthermore, the researchers classified the students’ clinical decision-making processes regarding VE and EFM in each scene into the following categories: ‘should do’, ‘should not do’, and ‘suspended’. The transcription symbols of recorded data from the group discussions are provided in Additional file 1 in the Supplementary Information section.

## Results

### Clinical decisions regarding VE and EFM

Table 2 shows the frequencies of conducting VE and EFM. The video groups decided to perform VE 16 times and EFM 20 times whereas the paper groups performed VE 23 times and EFM 25 times. In addition, the video groups chose not to perform VE eight times and EFM two times whereas the paper groups decided not to perform VE two times and EFM zero times. The frequencies of conducting VE and EFM in the video groups were below the commonly accepted standards of obstetrics in Japan. Conversely, the frequencies in the paper groups were above the standards. A qualitative analysis of group discussion showed that paper groups tended to decide more VE/FM.

**Table 2:** Decision-making on VE and EFM

Scene	Standard Practice*		Video groups												Paper groups											
			V1		V2		V3		V4		V5		V6		P1		P2		P3		P4		P5		P6	
1				■							×	×			●	■	×	■				■				
2		■			×	■									●	■	×	■		■						
3		■	●	■	●	■	●	■	×		●	■			●	■	●	■	●	■	●	■	●	■	●	
4	●		●	■	●	×	×	■	●	■	×			■	●	■	●	■	●							
5	●	■	×	■	×	■	●	■	●		×	■	●		●	■	●	■	●	■	●	■	●	■	●	■
6	●	■	●	■	●	■	●	■	●	■	●	■	●	■	●	■	●	■	●	■	●	■	●	■	●	■
	●3	■4	●16 ■20												●23 ■25											

*VE* vaginal examination, *EFM* electric fetal monitor

● Implementation of VE ■ Implementation of EFM × decide not to do VE or EFM

* VE based on general care of low risk women in Japan. EFM based on Guideline for Obstetrical Practice in Japan at the time of data collection in this study

### The students’ reflections on their clinical decision-making processes

This section includes qualitative content analysis and excerpts from the focus groups, especially the students’ perceptions regarding the influences of the case modality during each stage of clinical decision-making (Table 3).

**Table 3:** Characteristics of students’ clinical decision-making

Stage	Video case	Paper case
1) Cue acquisition	Psychosocial information, including that about family members Biased information gathering	Biomedical information on the pregnant woman and baby Systematic information gathering
2) Interpretation	Easy to imagine woman and family members Empathetic Psychosocially focused/ fragmented understanding	Difficult to imagine woman and family members Less empathetic Biomedically thorough/ sequential understanding
3) Evaluation/ decision-making	Woman- and family-centred holistic Practical and tailored, contextually sensitive care Refraining from monitoring and delayed decision-making	Healthcare provider-centred biomedical General, knowledge-driven care Frequent monitoring and quick decision-making

### Cue acquisition

#### Video groups

Psychosocially oriented information collection.

The students in the video groups found it helpful to imagine the case by grasping the context psychosocially. This also helped them understand the family as a whole, and they empathized with the woman and her family members even if they lacked clinical experience. (In the following excerpts, *V* indicates a video group, *P* indicates a paper group, number represents the group number, and the letter indicates the individual student.)

·*Through the video, we could see the entire picture, including the family members (V6-A).*

*· I empathized with the woman in labour, and I wanted to relieve her pain as quickly as possible (V3-C).*

*· It was easier to get into the situation, since it made me feel like I was playing an important role in it (V4-B).*

However, the students in the video groups felt a sense of urgency and had difficulty memorizing the information due to the rapid flow of details in the video. As a result, the discussion was narrowed down to specific and significant events that were somewhat emotionally biased.

*· In the video, I was unable to think slowly (V6-A).*

*· I felt a sense of urgency; I had to hurry if I were to do something, which also made me feel impatient (V6-D).*

*· Since I could not remember the information (in the previous scene), we simply discussed the problems in the current scene (V2-A).*

#### Paper groups

Biomedically oriented information collection.

In contrast, the students in the paper groups focused more on the biomedical data about pregnant woman and fetal heart rate. Since they could read the textual information repeatedly, they found it easy to remember the previous scene systematically.

*· I was able to focus on the quantitative data and abnormal data (P5-B).*

*· It was easy to compare the woman’s current and previous status as well as refer to her basic information, birth plan, and VE results, since all of the data was in writing (P6-C).*

However, the students in the paper groups, especially those that lacked experience in observing childbirth, found it difficult to imagine the situation of the woman and her family members. They also had difficulty grasping psychosocial aspects such as the feelings/emotions of the woman and her family members.

*· Since I had no experience in observing labour, it was difficult to imagine what it must be like (P3-B).*

*· Although the conversations were written in the paper case, I did not know how they were feeling. In other words, it was difficult for me to understand the feelings of the woman and her family members (P3-D).*

### Interpretation

#### Video groups

Easy to imagine woman holistically but a fragmented understanding.

In the video groups, the audio-visual information helped the students understand and empathize with the woman and her family members holistically and made them feel like ‘real attending midwives’.

*· Since the information was visual instead of textual, I was able to understand the personalities of the woman and her family members in a more detailed manner (V1-A).*

*· By watching the video, I could imagine myself as a person (in the scene) (V3-A).*

However, the students in the video groups felt that their biomedical assessment of the case was insufficient and superficial, which caused their assessment to become fragmented.

*· The assessment itself became somewhat superficial (V2-A).*

*· We skipped the assessment and only discussed the birth plan (V2-D).*

*· Since the video included a rapid flow of information, I was unsure whether my assessment was sufficient, and I wondered if my judgment was correct (V5-C).*

#### Paper groups

Biomedically sequential understanding but difficult to imagine woman holistically.

The students in the paper groups felt that it was easy to assess the case by analysing the information thoroughly and sequentially. They relied on biomedical and numerical rather than psychosocial information.

*· I was able to assess the woman sentence-by-sentence (P1-B).*

*· Since I was able to understand the numerical information as one criterion for judging the labour progress, I wanted to perform the VE (P4-A).*

However, it was difficult for the students to understand the woman holistically and to empathize. As a result, they monitored the woman frequently according to their own anxiety and biomedical viewpoints.

*· There was no image at all, so we decided to perform the VE (P1-C).*

*· We decided to perform EFM every time without considering whether the timing was right, since we were anxious about fetal dysfunction (P2-B).*

### Evaluation/decision-making

#### Video groups

Woman-centred practical and tailored care but restraint from monitoring and delayed decision-making.

The students in the video groups found it easy to consider when and how to administer individually tailored care. The planned care was contextually sensitive, woman-centred, and holistic. However, the students refrained from frequent monitoring.

*· It also made it easier to think about what type of care to provide and when and how it should be provided (V3-A).*

*· If the timing of EFM is poor, then it can be painful for the woman. In other words, we should never conduct EFM without considering the woman’s situation (V6-A).*

However, the students in the video groups regretted delaying the timing of VE and EFM. They also felt that they had over-considered the comfort of the woman and her family members and became emotionally involved in the case.

*· I over-focused on relieving her pain and caring for her family, so I missed the overall point (V2-D).*

*· Since VE can be painful, I wondered if I should stop doing it. I decided to perform (VE) later, but I was surprised that the cervix was almost fully dilated (V2-A).*

#### Paper groups

Healthcare provider-centred medical safety-oriented care but general, knowledge-driven care.

The students in the paper groups decided to perform VE and EFM quickly, and they did not focus on the woman’s comfort. The planned care was nonspecific, medical safety-oriented, and driven by textbook-based knowledge. Consequently, monitoring occurred frequently. Moreover, the students had trouble considering how to implement the planned care from a practical viewpoint.

*· We decided to perform EFM every time without considering whether the timing was right, since we were anxious about fetal dysfunction (P2-B).*

*· To be honest, we decided to perform VE and EFM at the same time (P2-A).*

*· There were various opinions regarding how to alleviate pain, but it was difficult to think about certain priorities such as identifying what was suitable for the woman, the timing, and the situation (P6-D).*

### Characteristics of clinical decision-making in the group discussions during CBL

The characteristics of the students’ clinical decision-making processes are also clarified in the group discussions in accordance with the characteristics in Table 3. The representative excerpts from the video and paper groups are included in Additional file 2 in the Supplementary Information section.

### Woman- and family-centred care by empathetic midwives

The attitudes, manners of speaking, and remarks differed between the video and paper groups. The students in the video groups exhibited more empathetic behaviours toward the woman. This was supported by their usage of personal pronouns, such as ‘I’ and ‘we’, which implied that they had a sense of ownership as midwives. Conversely, the students in the paper groups used third-person pronouns, such as ‘he’ and ‘they’, in order to describe what occurred in the scenario (see Excerpt 1 in Additional file 2 in the Supplementary Information section).

The students in the video groups were immersed in the case. They discussed woman- and family-centred care with empathetic attitudes as if they were in front of the woman and her partner in real life. In Excerpt 1, they used empathetic phrases to cheer up the anxious woman, to reassure her about the increasing intensity of labour pain, and to explain the likely outcome of her labour. They also used empathetic phrases regarding her partner, such as ‘her partner looks upset’, and considered the partner’s fatigue.

The students in the paper groups enumerated textbook-based general care. However, they rarely discussed specific approaches and the timing of care in the given context. The attitudes were healthcare provider-centred and biomedically oriented. In Excerpt 1, although they were aware of the lack of a practical care plan, they only stated textbook-based principles of human care, such as labour pain relief in accordance with the woman’s request, mental support, and being beside her. The students were able to objectively determine that the woman’s anxiety would adversely affect her labour progress, but they only provided biomedical information, such as ‘we need to tell her the pain will get more intense than it is now’, without empathetic remarks.

### Psychosocially oriented practical and tailored care

The video groups employed an approach to practical and tailored care that considered the woman’s psychosocial aspects. The care plan was discussed from a holistic viewpoint. Excerpt 2 in Additional file 2 shows careful consideration of psychosocial aspects, including the woman’s daily life activities, her emotions, her partner’s opinions, and the support from her family members. The students also proposed that the woman take a walk to instigate the labour process. Subsequently, they decided to perform EFM as she was resting.

The paper groups generally focused on the woman’s biomedical aspects and knowledge-driven general care. In Excerpt 2, the paper group decided to perform EFM and a VE rather quickly, after which they requested the related biomedical information. There were fewer discussions regarding when and how to perform EFM and VE. In other words, the care plan in the paper groups was biomedically oriented and abstractive.

### Refraining from performing invasive care

The video groups generally refrained from performing VE and EFM in consideration of the woman’s comfort whereas the paper groups performed VE and EFM to obtain objective biomedical information. In Excerpt 3 in Additional file 2, after confirming the woman’s situation, the students in the video group judged the progression of labour according to the nature of her labour pain and proposed a VE. However, after accusatory statements were made regarding excessive VE, the students determined that the labour progression was slow based on the findings of the previous VE, elapsed time, and the interval between contractions, and the VE decision was cancelled.

Conversely, the paper groups focused more on the early detection of abnormalities. In Excerpt 3, all students in the paper group agreed to perform a VE and EFM immediately after a short discussion. They decided to measure the woman’s blood pressure out of worry over hypertensive disorders of pregnancy because of her father’s history of high blood pressure, and, subsequently, they decided to conduct EFM quickly without careful consideration.

## Discussion

### Influence of the case modality on the students’ clinical decision-making processes

This exploratory study first found differences in the three stages of clinical decision-making processes, i.e. cue acquisition, interpretation, and evaluation/decision-making, between the midwifery students in the video and paper groups. It was suggested that the video groups also chose to perform EFM and VE less frequently than the paper groups.

### The cue acquisition stage

The video groups focused on the woman’s psychosocial aspects and showed empathy with her situation, but their information collection was biased. The video modality placed the students in the ‘actual’ labour process, and it made them feel a sense of authenticity and urgency. These findings are in line with those of previous studies [22, 24, 33]. More specifically, the video clearly visualized the woman’s pain and distress, which, in turn, motivated the students to become more empathetic. In the present study, the video groups had more difficulty retaining information than the paper groups. Previous studies have also highlighted the difficulty of identifying and extracting relevant information from video materials [24, 34, 35]. Although significant life event shown in the five-minute video used this study is most likely retained in learners’ long-term memory, educators should be aware of their limited capacities of memorization and information retention when viewing video materials.

In contrast, the paper groups mainly focused on the woman’s biomedical aspects, and they might not have sufficiently perceived the woman’s emotional dimension or sense of urgency. The focus group showed that the students with limited clinical experience had difficulty in creating an image of her psychosocial aspects. A lower achievement of imaging was also reported when paper patients were used [16].

### The interpretation stage

In the video groups, the students’ interest in and interpretation of the woman’s significant event were psychosocial, and their assessments were often fragmented. They lacked a comprehensive picture of the woman’s labour process. These findings are supported by previous studies in which video cases disrupted learners’ critical thinking [20]. Fragmented assessments may be caused by psychosocially biased information.

Conversely, in the paper groups, the students’ interest and interpretation were biomedical, and their assessments were thorough and sequential. The paper groups emphasized the importance of discussing and interpreting the biomedical aspects of the woman’s labour process. Since group members had the case information sheet in hand, they were able to refer to such information at their own pace [24]. Students in the paper group in our study could read and discuss information sheets, which should be advantageous for information gathering and interpretation. However, the use of a paper case would still require detailed descriptions of the woman’s psychosocial aspects and her family’s background to imagine the woman holistically.

### The evaluation/decision-making stage

The video groups adopted a woman- and family-centred holistic decision-making approach that considered the woman’s comfort. This approach was reflected in the students’ active discussions regarding how the care could be tailored to the wishes of the woman. These results are in line with the previous studies [22, 36, 37]. The other important finding in the present study is that the video groups generally tended to refrain from performing frequent EFM and VE. The video might have influenced the students to focus more on the woman’s comfort and reconsider the need to excessively perform such procedures.

In contrast, the paper groups adopted a healthcare provider-centred biomedical approach that emphasized medical safety over the women’s comfort. They also viewed the case from an objective perspective. Moreover, the discussion data showed that the students tended to frequently choose to perform EFM and VE to determine whether the woman had any serious problems. Such behaviour might have been partly due to their anxiety caused by the lack of knowledge. Thus, educators should be aware of this possible risk when using paper cases.

### Pedagogical implications of using different modalities to improve students’ clinical decision-making skills

This exploratory study revealed that video- and paper-based teaching materials have their own strengths and weaknesses.

Video cases are not only valuable for developing students’ information-gathering skills before entering a clinical setting but also important for planning tailored care that considers the woman’s comfort. However, comprehensive assessments might be insufficient in video cases. The following are suggestions for faculty members’ effective facilitation of learning with video cases.

1. Make video clips short, preferably less than 5 min, considering information processing ability.
2. Encourage students to write down the information while viewing.
3. Provide sufficient time for information sharing by students after viewing.
4. Calm down students when they are too emotionally involved.
5. Set learning goals for students to discuss both psychological and biomedical aspects and medical safety.

On the other hand, paper cases can be valuable for training students to make comprehensive patient assessments, especially regarding biomedical information. The following are suggestions for faculty to facilitate learning with paper cases.

1. Describe psychosocial information of the case, such as facial expressions, voice tones, conversations, and emotions.
2. Encourage students to share a common image of a woman/patient.
3. Ask students to formulate both practical and personalized care plans.
4. Encourage students to think how to communicate with the people and gather information in a real clinical setting.

In our study, we chose paper and video cases because of high feasibility and popularity of these modalities; however, it would be necessary to conduct further research to compare new modalities, such as e-CBL [38, 39] and simulation-based scenarios [40].

### Limitations

This study includes several limitations. First, the sample was small, and the study was performed using a single case scenario in one country. Although the scenario was carefully created, the possibility of bias remains, thus limiting the study’s validity. Cultural differences of childbirth and family relations should also be considered. Second, we did not perform a statistical analysis due to the small number of participants, and the frequency of EFM/VE was judged qualitatively from student interactions. Follow-up studies should be conducted in the future including the long-term effects of case modalities on students’ clinical decision-making processes and on their performance in actual clinical practice.

## Conclusion

This study clarified the different influences of video and paper case modalities on the clinical decision-making processes of midwifery students. The students’ perceptions of the case, the cues recognized by the students, and the emotions felt by the students might depend on the learning modalities. The video case made the students consider the woman and her family from a holistic viewpoint, which fostered their ability to provide tailored care based on their comfort. The paper case made the students more healthcare provider-centred, which fostered their ability to make clinical decisions based on thorough biomedical assessments. Educators should be aware of strengths and weaknesses of video and paper case modalities in students’ learning for clinical decision-making.

## Supplementary information

**Additional file 1.** Transcription symbols.

**Additional file 2.** Excerpts of group discussions.

## Data Availability

The datasets generated and analyzed during the current study are not publicly available since the patients` confidential information are included, but are available from the corresponding author on reasonable request.
