# Supplementary material for: How does video case-based learning influence clinical decision-making by midwifery students? An exploratory study
Source: BMC Med Educ. 2020 Mar 6;20:67. doi: 10.1186/s12909-020-1969-0 (PMC7059388; doi:10.1186/s12909-020-1969-0)
Supplement: Supplementary file 1 — Additional file 1. Transcription symbols. [file 12909_2020_1969_MOESM1_ESM.docx]

**Additional file 1:**

Transcription symbols *

| Symbol | Name | Use |
| --- | --- | --- |
| [text] | Brackets | Indicates the start and end points of overlapping speech. |
| = | Equal sign | Indicates the break and subsequent continuation of a single interrupted utterance. |
| (# of seconds) | Timed pause | A number in parentheses indicates the time, in seconds, of a pause in speech. |
| (.) | Micropause | A brief pause, usually less than 0.2 seconds. |
| . | Period or down arrow | Indicates falling pitch. |
| ? | Question mark or up arrow | Indicates rising pitch. |
| >text< | Greater than/less than symbols | Indicates that the enclosed speech was delivered more rapidly than usual for the speaker. |
| <text> | Less than/greater than symbols | Indicates that the enclosed speech was delivered more slowly than usual for the speaker. |
| ° | Degree symbol | Indicates whisper or reduced volume speech. |
| underline | Underlined text | Indicates the speaker is emphasizing or stressing the speech. |
| ::: | Colon(s) | Indicates prolongation of an utterance. |
| (text) | Parentheses | Speech that is unclear or in doubt in the transcript. |
| ((italic text)) | Double parentheses | Annotation of non-verbal activity.  Transcriber’s comment or explanation. |

* Jefferson G. Transcription Notation. In J. Atkinson & J. Heritage (Eds), Structures of Social Interaction. New York, Cambridge University Press, 1984, pp. ix-xvi
