# Supplementary material for: How does video case-based learning influence clinical decision-making by midwifery students? An exploratory study
Source: BMC Med Educ. 2020 Mar 6;20:67. doi: 10.1186/s12909-020-1969-0 (PMC7059388; doi:10.1186/s12909-020-1969-0)
Supplement: Supplementary file 2 — Additional file 2. Excerpts of group discussions. [file 12909_2020_1969_MOESM2_ESM.docx]

**Additional file 2:　Excerpts of group discussions**

Excerpt 1: Woman- and family-centred care by empathetic midwives　(scene 5)

| **V2: Woman- and family-centered holistic approach**  **and empathetic decision-making** | | **P6: Healthcare provider- centered biomedical approach**  **and less empathetic decision-making** | |
| --- | --- | --- | --- |
| 5  6  7  8  9  10  11  17  18  19  20  21  22  23  24 | C: because her partner looks upset (.) I want to say to him (.) “you can do this for her”.  B: (he) seems to be really upset.  C: but the pregnant woman can express her feelings calmly. I want to tell her, “now your baby is coming down around here (.) it’s a bouncing baby.”  D: (5.0) her partner became restless (.) right?  C: he doesn’t seem to know what to do. I wonder if he’s tired because he has gently stroked her body for a long time. can her mother take his place?  D: why not?  C: her partner might also feel stressed.  ((Turn 12-16))  B: the pregnant woman is panicked.  C: labour pains caused her to panic because she said “I’m scared.”  B: [what can we do for her?]  D: [how can we encourage her?]  A: we can say, “increasingly intensive labour pain is a normal part of the process of giving birth to your baby (.) and is a good sign.” we can also say, “I understand this is scary.”  C: do you think it’s better to tell her the time ((*of childbirth*))? (in the video) she asked us “what do you mean by for a while?”  B: but (.) we can’t tell the exact time, [can we]?  A: [how about] telling her the next goal? =Like (.) we say “when labour pains occur at 1 to 2 minute intervals (.) we’ll go to the birthing room together.” if we said this (.) she would feel better. | 49  50  157  158  159  160  161  214  215  216  217  218  219  220  221  222 | B: because she said with fear, “if the pains get more intensive (.) I’m scared and can’t bear it” (.) I think the progress in labour may not readily occur (ha-ha).  D: we have to support her mentally.  ((Turns 51-156))  D: as for her anxiety, like “if the pain get more intense (.) I am scared” (.) what can we do for her?  B: we need to tell her “the pain will get more (ha-ha) intensive than now” and around which part of her body they will be painful?  C: yeah (.) [I think so.]  B: [but] (.) I’m not exactly sure what will happen to her.  D: we can suggest ways to relieve her labour pains (.) and try to do some of them for her together with her family? (3.0) we can try to meet her request (ha-ha).  ((Turns 162-213))  C: ((*The moment the midwife entered her room*)) she complained about her pain to the midwife. So (.), () (she) may feel strong anxiety.  A: [yeah]  B: [I see]. we need to fully explain this point.  A: [uh-huh]  C: [uh-huh] as far as we can (.) we’ll be beside her (ha-ha)  D: we will be beside her (.) won’t we?  C: yep yep (ha-ha)  D: I think it would be nice to have the same person.  B: being beside her is important. |

Excerpt 2: Psychosocially oriented practical and tailored care (scene 2)

| **V2: Psychosocially oriented practical and tailored care** | | **P1: Biomedically oriented general care** | |
| --- | --- | --- | --- |
| 67  68  69  94  95  96  97  98  99  100  101 | C: I guess this would be weak labour pain.  B: uh. (10.0 s) how about doing fetal heart monitoring?  A: to check whether her labour pain is severe or not (.) we can try to do monitoring for 20 mins at this stage. When her pain is starting to be relieved (.) should we do something for her? or let her take a rest? ((*the cervix is*)) 3 cm dilated (.), not changed a lot ((*since the last examination*)).  ((Turns 70- 93))  A: why not discuss the process of her labour?  B: when her partner goes home(.) we will encourage her to walk around.  C: when should we check the fetal descent and rotation ((*by VE*))?  B: ((*because the fetal head is still far*)) I doubt whether we can properly understand the state of fetal rotation ((*by VE)*)  ((Turns 95-97))  C: I wonder if the fingers would reach the baby’ head. When can we use ((*the monitor*))?  A: hmm (.) when is better? before lunch time (.) we can try it once. I think we need to do it before lunch.  B: OK (.) we can do that.  C: ((*to sum up*)) we’ll first explain the process of labour to her (.) and then ask her to walk around. after that (.) we’ll ask her to put on the monitor along with taking a rest. | 1  2  3  4  5  6  7  8  9  10  11 | A: even though the effacement has progressed (.) why isn’t the cervix dilated?  B: it might be because the internal os ((*hasn’t been opened yet.*))  A: I see.  B: but (.) should we use a fetal monitor?  A: yeah (.) why not?  C: after she has seen the doctor (.) we will use it.  B: ((*according to the medical examination*)) the labour hasn’t proceeded yet, not yet.  B: how about assessing the condition of the woman’s body?  A: she seems to be excited ((*about the baby being born*)) without fatigue. (3.0) so, we can do fetal monitoring.  B: it would also be better to get her vitals.  can we do that?  ((*all the group members: yes*))  B: can we get the data on ((*fetal*)) monitoring and vitals? ((*to the researcher*)) |

Excerpt 3: Refraining from conducting invasive care (scene 4)

| **V5: Refraining from conducting VE/EFM** | | **P2: Quick decision-making on VE/EFM** | |
| --- | --- | --- | --- |
| 6  7  8  9  10  11  12  13  14  15  16  17  18  19  20  21  22  23  24  25  26  27  28  29 | C: ((*in the video*)) she said she had a backache [didn’t she?]  A: [yes] she said that.  C: she said strongly that I have backache, didn’t she?=  A: =like(.) the pain goes to her bottom  B: in the case that it goes to her bottom, should we do VE?  C: VE again?  A: we don’t need VE at this time  B: no need? (.) or? (.) we may need it.  C: we’ve done VE too often, right?  D: I see. [at 1 pm (.) we also did it？]  C: [at 1 pm we did VE.]  B: when did we do VE?  A: as it’s obviously 4 cm dilated (.) I’m sure it will not proceed at this stage.  D: yeah  C: the last VE was done at 1 pm. Now, it’s 3 pm, so it just passed 2 hours.  B: I see.  C: actually (.) what is her condition ((*when the contractions continue*)) for 40 seconds?  A: 40 seconds.  C: the labour pains are still in 3 to 5 min intervals, so not yet. If it is in 2 to 3.5 min intervals. now it ((*the contraction*)) is still for 40 seconds.  A: yep  C: it doesn’t last a certain length of time.  B: I see.  C: still [a long way to go].  A: [yeah] we need to wait. | 45  46  47  48  49  50  51  52  53  54  55  56  57  58  59  60  61  62  63  64  65  66  67  68 | A: it’s better to do monitoring, and as she has started to push, we also need to [do VE].  C: [yeah] (.) it goes well.  B: vitals (.) should we take it every 4 hours?  A: this textbook says it can be every few hours. when labour pains are getting intensive (.) it sometimes causes some women’s blood pressure to rise.  B: oh [I get it].  C: [I see].  A: previously (.) she didn’t have any problems with blood pressure.  C: what about any problems during her pregnancy?  B: she didn’t have any.  A: (but) her father has high blood pressure.  C: her family history indicates it  B: because her family history indicates it°  A: so (.) in that case we can have [her vitals]  B: [her vitals]  A: right VE <and vitals and> (.) what about monitoring as well? we use it (EFM). We use it (EFM)?  C: when did we use it last time?  A: I think we’ll do the monitoring in the second phase.  B: we::ll (.) so we don’t have to do right now.  A: but we should do it one more time (.) should we do it? we can better understand the fetal condition.  B: O::K (.) well then.  C: we can also [do VE].  B: [VE and vitals] as well.  A: [yes I agree.]  C: [also monitor] ((*EFM*)) |
